# A tabular data generation framework guided by downstream tasks optimization

**DOI:** 10.1038/s41598-024-65777-9

**Published:** 2024-07-03

**Authors:** Fengwei Jia, Hongli Zhu, Fengyuan Jia, Xinyue Ren, Siqi Chen, Hongming Tan, Wai Kin Victor Chan

**Affiliations:** 1 Tsinghua Shenzhen International Graduate School, Tsinghua University, Shenzhen, 518055 People’s Republic of China; 2 Tsinghua-Berkeley Shenzhen Institute, Tsinghua University, Shenzhen, 518055 People’s Republic of China; 3 School of Mechanical Engineering, Anhui University of Technology, Maanshan, Anhui, 243032 China; 4 International Science and Technology Information Center, Shenzhen, 518055 People’s Republic of China

**Keywords:** Information technology, Scientific data

## Abstract

Recently, generative models have been gradually emerging into the extended dataset field, showcasing their advantages. However, when it comes to generating tabular data, these models often fail to satisfy the constraints of numerical columns, which cannot generate high-quality datasets that accurately represent real-world data and are suitable for the intended downstream applications. Responding to the challenge, we propose a tabular data generation framework guided by downstream task optimization (TDGGD). It incorporates three indicators into each time step of diffusion generation, using gradient optimization to align the generated fake data. Unlike the traditional strategy of separating the downstream task model from the upstream data synthesis model, TDGGD ensures that the generated data has highly focused columns feasibility in upstream real tabular data. For downstream task, TDGGD strikes the utility of tabular data over solely pursuing statistical fidelity. Through extensive experiments conducted on real-world tables with explicit column constraints and tables without explicit column constraints, we have demonstrated that TDGGD ensures increasing data volume while enhancing prediction accuracy. To the best of our knowledge, this is the first instance of deploying downstream information into a diffusion model framework.

## Introduction

Specific domain data, known as vertical domain, has become a key resource to drive innovation in various fields, such as healthcare, finance, and education. However, the insufficient amount of data has become a prominent problem when building and optimizing machine learning models in these fields. Vertical domain models require a large amount of labeled data to capture specific rules and patterns. However, due to the high cost of data acquisition and cumbersome labeling work, the amount of data is often difficult to meet the needs of model training. Insufficient data leads to limited accuracy, poor generalization, and an increased risk of overfitting. To overcome these challenges, researchers have proposed a variety of strategies, including data augmentation techniques to expand training samples, transfer learning to extract features using pre-trained models, and unsupervised learning to mine the intrinsic structure of data. These methods can alleviate the problem of insufficient data to a certain extent and improve the performance and application effect of the model.

The advent of synthetic data generation (SDG) provides a promising solution to address the limitations, lack of representation, or bias in datasets^1^. Synthetic data generation is a technique that creates new datasets mirroring the characteristics and structure of the original dataset. It has found widespread application in various fields, including data processing and machine learning. These artificial datasets offer advantages such as low cost, high controllability^2^.

Tabular data generation (TDG) is a subset of synthetic data generation that specifically targets the creation of new tabular datasets^3^. The aim is to generate new tabular data that are representative of the original tabular data and maintain the underlying structure and relationships between different pieces of table. It is particularly useful in scenarios where the original dataset is extremely scarce resources, which limiting the scope and effectiveness of machine learning models, such as rare case related data in medical data, complex ship design parameters.

The advancement in TDG techniques has opened up new possibilities for data-driven applications in various fields, such as financial modeling^1^, healthcare analytics^4^, and scientific research^5^. By leveraging synthetic datasets generated through TDG, these applications can now train more accurate and robust machine learning models despite limited or biased real-world data.

The advanced techniques such as generative adversarial networks (GANs)^2,6,7^ and diffusion propobility networks^3,8–10^ have also been employed in TDG to generate more diverse and realistic tabular data. These techniques have enabled the generation of synthetic datasets that are not only representative of the original dataset but also possess higher levels of complexity and realism.

Despite these advancements, a pivotal challenge persists. A core impediment we encounter is the disparity between the data requirements of downstream tasks and the impact of synthetic data on their analysis. Merely generating fake data that superficially resembles real data does not inherently guarantee its non-interference with downstream analyses. Specifically, the column constraints of the real tabular data, ensuring that the generated fake data remains consistent in column constraints and content with the real data. Therefore, we assume that solely focusing on the similarity between generated data and real data is not conducive to downstream task’s responsibility. Instead, we should pay more attention to the impact of generated data on downstream task analysis.

For example, in the task of generating tabular data for hull parameter generation, if the generative model does not consider downstream tasks (such as whether the hull parameters meet the constraints of the hull having no voids), then even though the numerical distribution range of the single column data is similar to that of the real data, it fails to construct a three-dimensional hull that is usable by humans when multiple columns collectively form a unified hull. Therefore, it is necessary for such tasks to generate more practical tabular data tailored to the requirements of downstream tasks.

To imporve the generated data utility, we propose a general TDGGD framework. Specifically, to address the significant differences in the numerical distribution between fake data and real data, we adopt an improved and simplified indicator (Easy Indicator, EI) that can determine the authenticity of the generated fake data. To tackle the issue of low feasibility constraint between fake data columns, we introduce multiple modified fuzzy indicators (Ambiguous Indicator, AI), which can implicitly learn the constraint relationships between columns. Moreover, to mitigate the impact of fake data on downstream task accuracy, we incorporate multiple performance indicators (Hard Indicator, HI) to determine the fake data for key columns according to the requirements of downstream tasks. The main contributions are the following:

1. Novel diffusion model framework which introduces downstream task targets to improve its utility on generated fake data.
2. Efficient modeling of table constraints via easy indicator, ambiguous indicator, and hard indicator to satisfy feasibility of generated fake data, including focusing on key columns, adhering to constraint conditions, and enhancing prediction accuracy.
3. Providing an effective solution on tabular data scarcity for datasets augumentation.

### Related work

### Diffusion models

The first diffusion models can be attributed to research^11^, who proposed the iterative improvement of Gaussian noise data vectors in multiple time steps to transform random data into data that reflects the statistical characteristics of the training data. Building upon this work, subsequent advancements led to the development of the Denoising Diffusion Probabilistic Model (DDPM)^12^.

Following DDPM, numerous researchers have conducted studies based on diffsuion foundations. Diffusion models have demonstrated advantages over the popular generative model GAN^13^, particularly in terms of coverage and diversity of generated data, rendering sampling time negligible in specific contexts. In order to further improve computation time, techniques such as Denoising Diffusion Implicit Models (DDIM)^14^ and Latent Diffusion Models (LDM)^15^ have been developed, which enhance sampling efficiency by providing greater tolerance to the Markov process. As for fake data quality, DDPM-based models can specify the direction of generated data by incorporating gradient-guided guidance information from an additional classifier neural network^16^. This is particularly beneficial in tasks such as image generation, where the desired label category of the generated images can be determined based on human preferences. Moreover, the idea of utilizing additional guidance information has been extended to other domains, including text-to-image generation^17^ and image-to-3D conversion^18^.

Diffusion models have demonstrated advantages not only in computer vision but also in natural language generation^19^, robust learning^20^, temporal data modeling^21^, multi-modal learning^22^, molecular graph modeling^23^, and other fields. Multiple survey articles^24–28^ are constantly emerging, showcasing the growing recognition of diffusion models in themselves domains. DDPM has established itself as a widely adopted base model, occupying a prominent position in data generation. It excels in generating high-quality synthetic data, handling complex constraints, and producing accurate outputs under proper guidance, making it an exceptional deep generation model for tabular data.

### Generative models for tabular data augmentation

Tabular data generation is gaining prominence as a popular modality for creating synthetic data^29^. Initially, Variational AutoEncoders (VAEs)^30^ were the dominant framework, wherein GOGGLE^31^ employed a structure-based learning approach to model tabular data, while also regularizing variable dependencies to mitigate overfitting on smaller datasets. Subsequently, GAN-based methods^32,33^ have emerged as a foundational framework due to their capability to effectively model data structures and generate new attack vectors. CTGAN^34^ introduced a novel conditional generative adversarial network, incorporating a classifier to provide additional supervision, thereby enhancing its applicability in machine learning contexts. CTGAN-Conv1D^6^ combines two architectures - conditional attribute generative adversarial networks and 1D convolutional architecture, effectively capturing various facets of the desired output and generating realistic samples. More recently, diffusion-based methods have been explored, such as TabDDPM^3^, which integrates Gaussian and multinomial diffusion models, along with quantile transformer and one-hot Encoder superimposed vectors to synthesize mixed-type tabular data. ResBit^35^ underscores the preprocessing of tabular data, implementing bit compression for discrete data to improve diffusion efficiency. AutoDiff^36^ situates the diffusion model between the encoder and decoder, exclusively generating the latent representation. Furthermore, it categorizes data into numerical, binary, and categorical types based on frequency, and introduces a frequency variable to determine whether to replace new values. TableDiffusion^37^ incorporates differential privacy stochastic gradient descent into the training process, validating the privacy protection of mixed-type synthetic tabular data.

Tabular data generation is also widely used in downstream fields. In the economic domain, FinDiff^1^ introduces normalization of numerical data and categorical embedding to obtain preprocessed input tabular data, restoring the original data space, thereby ensuring the security of banking data. In the medical domain, EHR-Safe^38^ synthesizes electronic health records, particularly addressing highly-varying sequence lengths for time-varying features. In the engineering domain, ShipGen^5^ generates constraints that meet ship design parameters, while visualizing the engineering tabular data of ship structures, resolving time-consuming and inefficient issues in ship design.

To the best of our knowledge, the TDGGD is the initial endeavor to improve a diffusion model for focusing on prediction accuracy in the downstream task, rather than measuring similarity between real data and fake data.

## Motivation

During the training of the diffusion model, the original DDPM^12^ training loss of the neural network is exclusively utilized for predicting the random Gaussian noise injected in the forward diffusion process. Introducing additional alterations does not impact the resulting Gaussian distribution. However, in the DDPM sampling process, the loss influences the distribution of intermediate hidden variable data at each time step. It leads to a cumulative enhancement in the fidelity of the ultimately generated data.

From the perspective of parameter optimization of neural network models, we have made innovative adjustments to the loss value of the diffusion model’s time step *t*. Specifically, we have introduced a regularization term into the original loss value of the diffusion model, which aims to promote downstream tasks. In this way, the loss function can be represented as:

$$\begin{aligned} \text {Entire Loss} = \text {Loss (DDPM)} + \text {Regularization} \end{aligned}$$

where Loss (DDPM) is used to improve similarity, and regularization term add complexity.

Without regularization, the model may focus exclusively on minimizing the DDPM loss, potentially leading to overfitting and a decrease in the quality of generated data. This occurs because the model may learn to produce outputs that closely match the training data but fail to generalize well to unseen examples. Under regularization, the model is encouraged to explore a wider range of solutions, allowing it to find a balance between minimizing the DDPM loss and satisfying the regularization constraints. Although the increase in reasonableness and generalizability often outweighs this minor loss of precision, it is worth to improve data utility.

Consequently, the TDGGD framework encompasses three indicators to guide the model in generating data in various directions. By adding these indicators, the model’s optimization direction has become clearer, reducing the oscillation between training and validation errors. This allows us to find an optimal model strategy for better model performance.

## Learning objective

By training the model on real data *X*, we get the target *Y*. To augment dataset, we generate a fake data $X'$, and ensure that there is a significant difference between *X* and $X'$ under same feasibility. If *X* and $X'$ were too similar, they would be indistinguishable to downstream tasks, rendering the entire process meaningless.

Then, we share $X'$ with downstream scholars and practitioners, and they use $X'$ to get the prediction, which is marked as $Y'$. The goal of the experiment is to ensure that *X* and $X'$ are as different as possible, while trying to make *Y* and $Y'$ consistent. In the experimental part, we will conduct in-depth analysis of the feasibility and coverage of fake data of $X'$, and use the prediction target $Y'$ and calculated target $\tilde{Y'}$ for detailed evaluation.

When analyzing the numerical characteristics of training data, we adopt two ways to achieve the target value: $Y'$ is model inference of downstream tasks by regression model prediction, which is more common in machine learning tasks; $\tilde{Y'}$ is model calculation of downstream by by using the classic simulation analysis, i.e., by formula, which is more common in operations optimization tasks. However, we must note that most difficult tasks cannot clearly obtain the formula of the predicted target.

## Method

### Architecture

**Figure 1:**
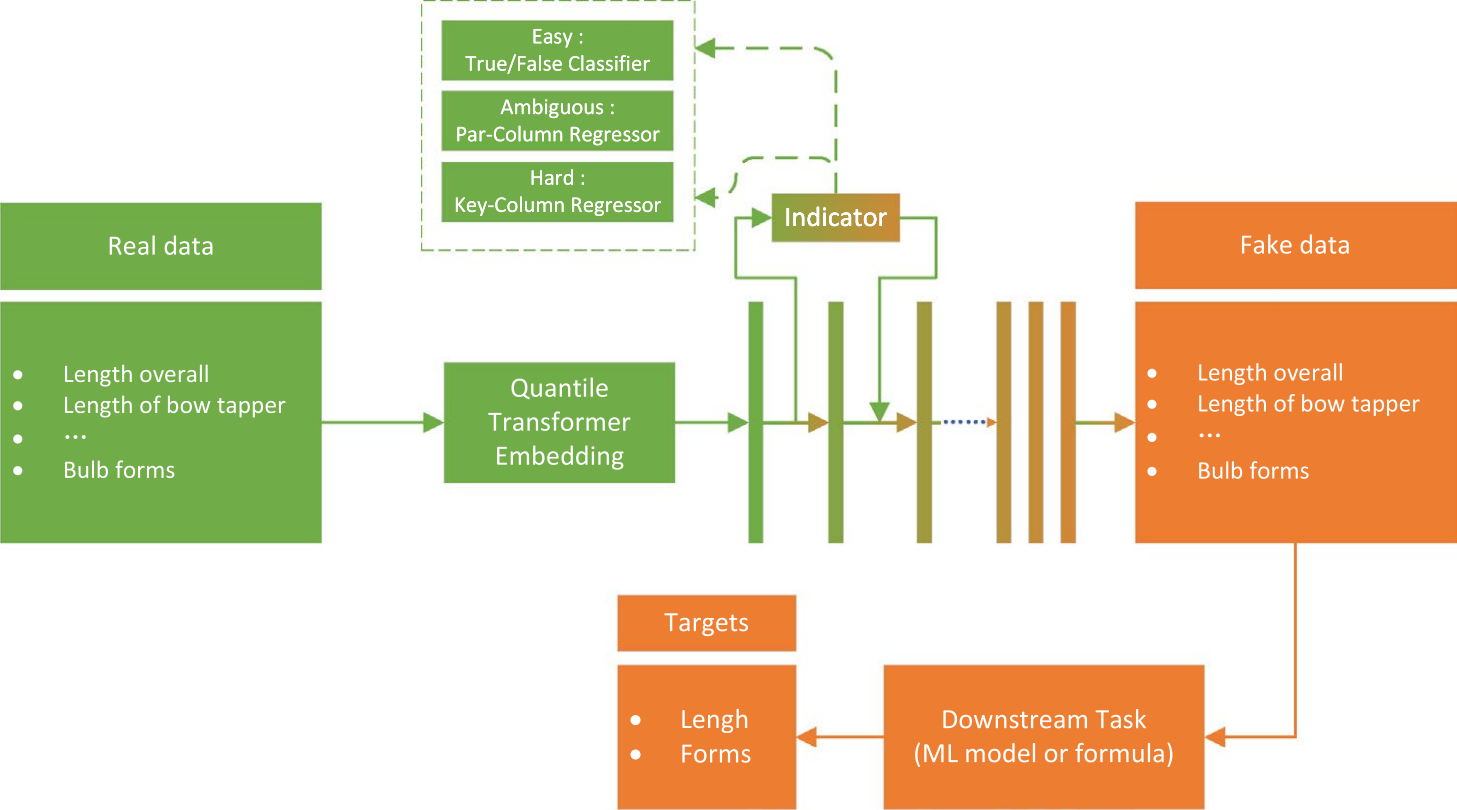
The overall framework for Tabular Data Generation Guided by Downstream Task Optimization. The green parts are real data and orange parts are fake data.

The TDGGD framework as a whole is illustrated in Fig. 1. It comprises four main modules, each dedicated to addressing specific issues in tabular data generation tasks. The subsequent subsections will delineate the key components. The specific parameters’ configurations are shown in Supplementary Tables S1–S4.

1. *Denoising Diffusion Probabilistic Models (DDPM)*: The main pipline generate similar tabular data by utilizing a Markov chain and probabilistic denoising.
2. *Easy Indicator (EI)*: EI modules involves using a binary classification method to evaluate and adjust the generated fake data. EI allows the generated fake data to retain certain structural characteristics of the original real data. At the same time, the generated table data approximates the original data, enabling downstream models to better understand the data and improve the accuracy of predictions.
3. *Hard Indicator (HI)*: HI modules involves identifying key columns based on the requirements of the downstream tasks and then generating fake data that meets these requirements through target optimization and gradient guidance. HI ensures that the generated table data fulfills the needs of downstream tasks while maintaining data logical consistency.
4. *Ambiguous Indicator (AI)*: AI modules implicitly learns the constraints between columns, using these constraints to ensure that the generated fake data complies with the inter-column relationships. AI can automatically handle the constraints of the original data, making the generated fake data more aligned with real-world situations and logical requirements.

### Denoising diffusion probabilistic mdels

The Denoising Diffusion Probabilistic Models (DDPM)^12^ is a generative model that learns to reverse the process of adding noise $\epsilon$ to tabular data *X*, effectively transforming random noise back into realistic samples $X'$ drawn from a target distribution by utilizing a Markov chain and probabilistic denoising techniques.

The forward process $q\left( x_{1:T} | x_{0}\right) {=}\prod _{t=1}^Tq\left( x_t | x_{t - 1}\right)$ gradually adds noise to an initial sample $x_0$ from the data distribution $q\left( x_0\right)$ sampling noise from the predefined distributions $q\left( x_t | x_{t - 1}\right)$ with variances $\left\{ \beta _1, ..., \beta _T\right\}$.

The reverse diffusion process $p\left( x_{0:T}\right) {=}\prod _{t=1}^Tp\left( x_{t-1} | x_{t}\right)$ gradually denoises a latent variable $x_T{\sim }q\left( x_T\right)$ and allows generating new data samples from $q(x_0)$. Distributions $p\left( x_{t - 1} | x_t\right)$ are usually unknown and approximated by a neural network with parameters $\theta$.

The DDPM used as the main pipeline to generate tabular data,which is inspired by the work^3^. The entire algorithm description can be found in supplementary material.

1 $$\begin{aligned} X_{t-1} =\frac{1}{\sqrt{\alpha _t}} \left( X_t - \frac{1 - \alpha _t}{\sqrt{1 - \bar{\alpha }_t}} \epsilon _{\theta }(X_t,t)\right) + \sigma _t(Z(1-\gamma )) \end{aligned}$$

### Easy indicator

A significant challenge in tabular data generation is achieving high fidelity of fake data. The Easy Indicator (EI) employs a binary classification of “True or False” samples to ensure that the generated data retains certain structural features of the original real data, such as temporal trends and periodic changes. At the same time, the generated tabular data approximates the original data, enabling downstream models to better understand the data and improve prediction accuracy.

In more detail, for real tabular data $X \sim Restrict([c_j])$ with “True” label of 0, construct a similarly scaled set of values $\tilde{X}$ not satisfying the constraints $Restrict([c_j])$ with “False” label of 1. Randomly merge *X* and $X'$ to form the new data, corresponding outputting labels . In each diffusion iteration, randomly extract batches of rows $[r_1,r2, \ldots ]$ as the EI classifier input data. EI uses a simple MLP architecture^39^, the process is as follows:

2 $$\begin{aligned} \begin{aligned} MLPBlock(X, X')&= Dropout(ReLU(Linear(X,X'))) \\ Label[0,1]&= \{EI(X, X') | Linear(MLPBlock(...MLPBlock(X,X'))) \} \end{aligned} \end{aligned}$$

The model uses binary cross-entropy to calculate loss and Adam with weight decay regularization^40^.

Moreover, when the downstream task is binary classification, EI is useful for handling data with clear classification properties. For example, boolean column labels are typically representative of classification columns, being intuitive and easy to understand. In downstream classification models, the EI helps to judge and predict category attributes. EI selects features closely related to the downstream task through these indicators and uses them as input data to train classification models. Therefore, classification columns directly related to the downstream task can be used to train classifier models without needing to consider dropping certain columns.

### Hard indicator

A second challenge in tabular data generation is ensuring the high efficiency of downstream tasks. The Hard Indicator (HI) determines the key columns based on the requirements of the downstream task, and then generates synthetic data that meet these requirements through target optimization and gradient guidance.

Specifically, for real tabular data *X* with target $Y=[Y_h]$, construct *h* residual neural networks with *h* target columns. HI adopts the residual MLP architecture^13^. For a tabular input *X* at timestep *t* with regression label *Y*, the process is as follows:

3 $$\begin{aligned} \begin{aligned} t_{embedding}&= LinearBlock(SinTimeEmb(t)) \\ y_{embedding}&= LinearBlock(Y) \\ y_{embedding}&= \{HI(X,t) | MLP(X) + t_{embedding}\} \end{aligned} \end{aligned}$$

**Figure 2:**
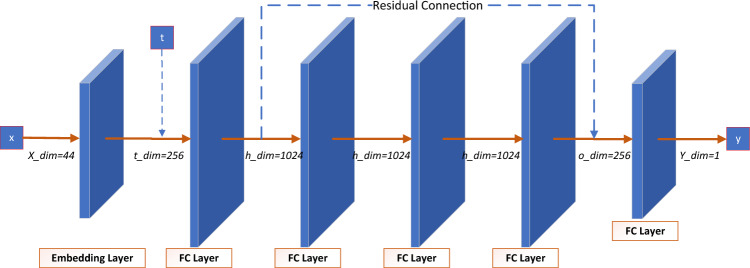
The structure of hard indicator.

The MLP structure of the residual connection is shown in Fig. 2. HI uses Mean Squared Error (MSE) to calculate loss and the Adam with weight decay regularization^40^.

Additionally, when the downstream task is numerical regression, HI helps to filter out features that significantly influence the prediction results of the downstream task, i.e., implicitly expressed key columns, and uses these features as input to train HI. For features not closely related to the current task, HI selectively discards them or assigns them lower weights, reducing model complexity and the risk of overfitting.

**Figure 3:**
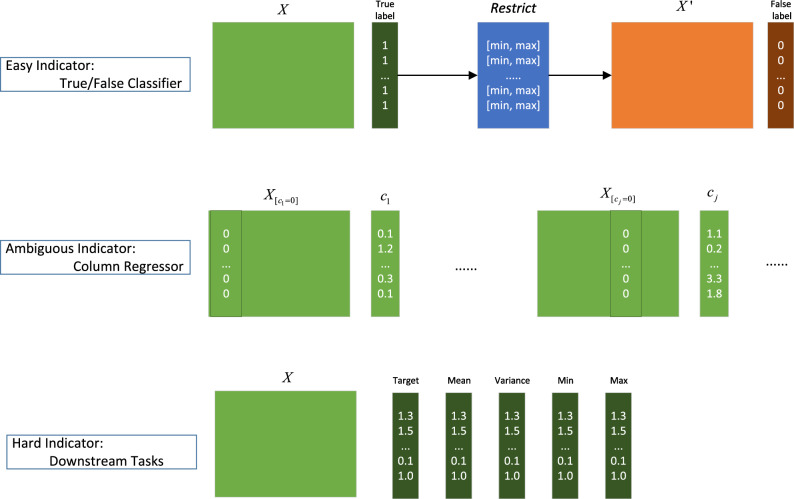
Data preparation. The green parts are real data and orange parts are fake data.

In the experiments, for datasets with only one column for the downstream task, such as the California House dataset, we additionally expand to five downstream task columns as shown in Fig. 3. By predicting the maximum, minimum, average, and variance of the target column, we obtain information about data distribution and central tendency. This information is crucial for understanding the overall characteristics of the data and for subsequent analysis.

### Ambiguous indicator

Another challenge in generating tabular data is the numerical relationship constraints between column values in the table. The Ambiguous Indicator (AI) implicitly learns the constraints between columns, using these constraints to ensure that the generated table data satisfy the inter-column relationships. We can divide all columns of *X* into two categories: a set of columns $c_j^u$ unrelated to the current column $c_j$, and another set of columns $c_j^r$ that have potential relationships with the current column $c_j$, where $u + r +1 = n$. These potentially related columns $c_j^r$ have an ambiguous impact on downstream tasks, where discarding them might affect prediction accuracy. Therefore, the output of the regression model is a randomly selected subset of columns $c_j$ as conjectured features, while the input is all column vectors, with the *j*-th column filled with zeros.

In more detail, for real tabular data *X* with $x_j=0$, construct *n* regression networks. For a tabular input *X* at timestep *t* with regression label $c_j$, they are processed by the residual MLP architecture, similar to the hard indicator.

4 $$\begin{aligned} \begin{aligned} t_{embedding}&= LinearBlock(SinTimeEmb(t)) \\ X_{AI}&= [c_1, c_2,.., c_j=0, c_{j+1}, c_n] \\ X_{j, embedding}&= {AI(X,t) | MLP(X_{AI}) + t_{embedding}} \end{aligned} \end{aligned}$$

The residual connection structure is shown in Fig. 2. The loss is calculated using MSE and the model weights are optimized with the the Adam with weight decay regularization^40^.

Additionally, AI’s prediction after filling $c_j = 0$ helps distinguish potential missing values or incomplete data. Considering the integrity and consistency of data, it ensures that the generated data $X'$ does not negatively impact overall data analysis. This not only improves the accuracy of data analysis but also greatly reduces the burden of manually handling missing values.

### Entire procedure

The three aforementioned indicators play a key role in different dimensions of the tabular data during the classification in EI, regression in AI, and evaluation processes in HI. By selecting appropriate types of indicators and algorithm optimization methods, we can more effectively achieve the goals of downstream tasks and significantly enhance the overall performance of the model.

During training Algorithm 1, DDPM is the main pipeline process, which handles the real and feasible tabular data *X* with quantile normalization and random noise given the timestep embedding. MSE is used as the model loss function to compare the difference between the noise removed by the reverse process and the noise added by the forward process. The training procedure similar with DDPM can be found on Supplementary.

During sampling Algorithm 2, the gradients of EI, HI, and AI are used to improve the DDPM’s sampling process. Following the multi-objective optimization theory of Pareto optimization, we introduce the hyper-parameters $\lambda$ to normalize the multiple indicators, which $\lambda =0.5$ suggested in research^5^. We scale the HI and AI vectors to positive values and sum them to 1 with $\gamma$. The sampling process can be formulated as follows:

5 $$\begin{aligned} \begin{aligned} X_{t-1} =&\frac{1}{\sqrt{\alpha _t}} \left( X_t - \frac{1 - \alpha _t}{\sqrt{1 - \bar{\alpha }_t}} \epsilon _{\theta }(X_t,t)\right) + \sigma _t(Z(1-\gamma )) \\&+ \gamma \nabla _{X_t}EI(y|X_t) + \frac{1}{n}\sum \limits _{i = 1}^n {{\nabla _{X_t}}} AI({C_i}|{X_t}) - \sum _{j=1}^{h}{\lambda _i\nabla _{X_t}HI(H_j|X_t)} \end{aligned} \end{aligned}$$

In practice, it is crucial to correctly select and apply these indicators. Easy indicators, while simple and intuitive, may not fully capture the relationships between complex data; ambiguous indicators, although capable of revealing hidden data relationships, might increase the uncertainty in the model; hard indicators help improve the accuracy of the model but may lead to excessive complexity. In the future, we need to choose indicator types and adjust model strategies based on specific problems and data characteristics.

**Algorithm 1:**
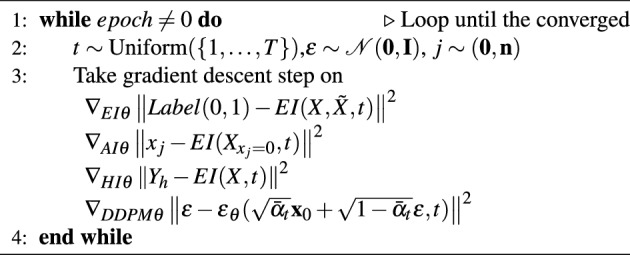
Training Algorithm.

**Algorithm 2:**
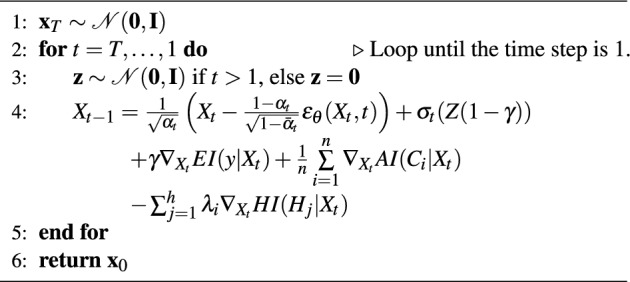
Sampling Algorithm.

## Experiments

All experimental results were conducted on a laptop running Windows 11 environment, with 16GB memory, an NVIDIA GeForce RTX 4060 Laptop GPU, and a 13th Gen Intel(R) Core(TM) i7-13650HX CPU.

### Description of the datasets

The cases analysis focuses on a common machine learning dataset “California Housing”^41^ without explicit column constraints and a specific downstream task “Ship-D”^42^ with clear column constraints . The original California Housing data is fetched from the sci-kit learn library, and the Ship-D dataset is obtained from the authors’ shared links. Both are tabular datasets with at least one regression target column, for which we use the rest of the columns to fit our model.

The **California Housing**^41^ dataset is an open-source dataset for regression problems. It contains 20,640 samples and features across 8 dimensions. All features are of real number type, and the target column “MedHouseVal” is also a real number, ranging from 0.15 to 5. This dataset is designed to help machine learning algorithms predict house prices in different regions of California.

The **Ship Parameters Datasets (Ship-D)**^42^ is from the MIT DeCoDE Lab, consists of 30,000 samples. The dataset contains 44 features for hull parameters and calculates 7 metrics. These 7 performance metrics describe the quality of a hull, considering the hulls’ hydrodynamics, hydrostatics, and manufacturability. The detailed calculation formula can be found in the original paper. We note the corresponding symbol representations in Supplementary.

### California house data preparation

We utilized the common California House dataset for regression tasks, where the downstream task is solely to predict the target column values. Here, we added an additional set of 4 tasks as supplements to the downstream predictive task with a single-column target, that is, maximum, minimum, mean, and variance. There are sufficient reasons to consider these as representative of potential future downstream task computations.

For preprocessing the input feature vector *X* of the original dataset, we constructed the feature vector $X'$ required by the EI, which does not meet certain conditions. For the Ambiguous indicator, the input $X_{[{c_j} = 0]}$ and the output $c_j$ are set up. The Hard indicator involves the input feature *X* and five downstream predictive column vectors *Target*, *Mean*, *Variance*, *Min*, *Max* respect to original target, maximum, minimum, mean, and variance.

Through the above data preprocessing steps, we can obtain richer and more accurate feature representations and provide the model with multiple targets.

### Compared methods

As the first method to apply diffusion models to synthetic tabular data, TabDDPM^3^ merges the continuous space Gaussian diffusion model and the discrete space polynomial diffusion model in a cascading manner. The TabDDPM design a combined loss values by mean summation within predictive noise neural network model. To compared the performance, these are five methods used in following experiments: RTVAE^43^, CTGAN^34^, TabDDPM^3^, DDPM with classifier^16^ (EI), ShipGen^5^(EI+HI). For fairness, the method is coded using the Pytorch library and maintain the default hyperparameters shared by the authors.

### Training performance

**Figure 4:**
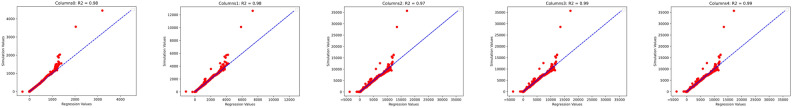
Comparison of the downstream prediction *Y* and $Y'$ across the California House datasets with the multiple targets. A perfect prediction (R2=1) is shown by the blue dashed line. The columns from 0 to 4 represent downstream target [’mean’, ’std’, ’max’, ’min’, ’MedHouseVal’] columns. Among them, ’MedHouseVal’ column is the target of downstream task prediction, while the others are constructed task targets.

The tabular data from the Ship-D and California House datasets were used to train AI and HI residual neural networks for predicting a target variable. The results of the training have been summarized in Supplementary Tables S5 and S6, which indicated by the R2 score as a measure of goodness of fit,. All the indicators are approximately regressed to best fitness, nearly one R2 score. Especially, the EI is the classifier on binary cross entropy and get 1.0 F1 score, which enable EI in TDGGD sampling with these columns. Additionally, it is mentioned that Fig. 4 displays the entire plots of the regression prediction versus the simulation calculation for the Hard indicator. The blue dashed line in the figures represents the perfect regression prediction, aligning with the simulation calculation. It is noted that all the neural networks had high R2 fits and closely hugged the blue dashed line. The training of neural networks resulted in high-quality fits based on R2 values and results in close alignment with the blue dashed line in the plots, indicating accurate regression predictions.

### Generation performance

Unlike the papers of TabDDPM^3^, which adopts simple machine learning methods to predict the utility of fake data, we pay more attention to considering downstream task to ensure the feasibility and coverage of generated data as Table 1. The meaning and calculation method of each metrics will be explained in Supplementary.

**Table 1:** Evaluation metrics in experiments section.

Compared variables		Evaluation metrics		
*X*	$X'$	Feasibility rate	DCR,NNDR	kAnonymization, lDiversityDistinct ,kMap,DeltaPresence, identifiabilityScore
*X*, *Y*	$X',Y'$	Coverage	Realism	Normalized Both
*Y*	$Y'$	Scaled factor		

#### Visual analysis

**Figure 5:**
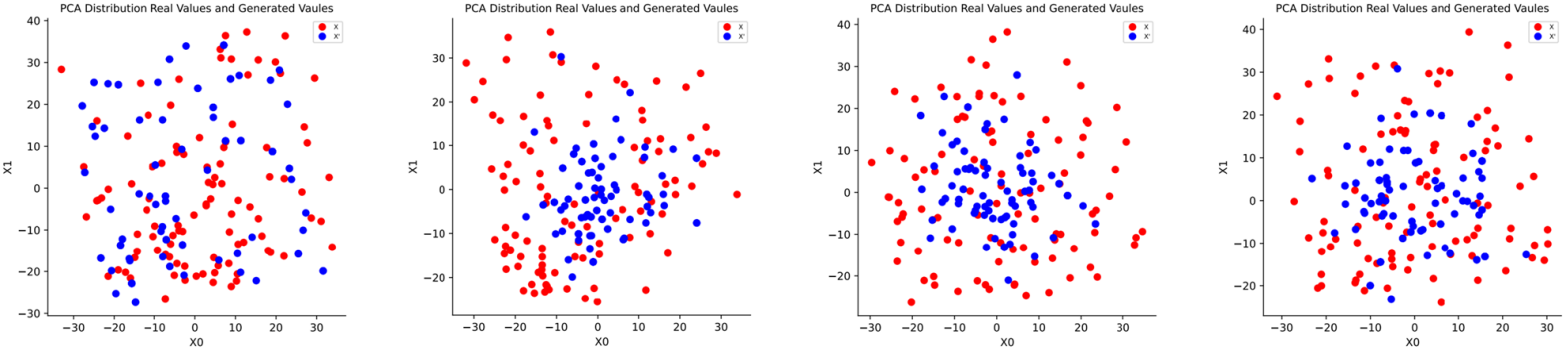
Two-dimensional principal component analysis of the Ship-D dataset reveals that the generated data using a standard DDPM maintained most of the dataset coverage. From left to right, the strategies corresponding are Base, EI, EI+HI, and EI+HI+AI.

A two-dimensional principal component analysis (PCA) was performed on the Ship-D dataset to visualize the distribution of generated fake data in comparison to the original data. Figure 5 refering to Table 3 are maintained most of the dataset coverage, with normalized coverage ratios of 0.984 for the base strategy, 0.969 for the EI strategy, 0.969 for the ShipGen, and 0.9699 for the TDGGD. These results suggest that the TDGGD generated data closely matches the original dataset in terms of coverage, as reflected by the high normalized coverage ratios.

#### Feasibility analysis

The feasibility metrics evaluate whether the generated data adheres to the laws of the real world. For instance, in the Ship-D dataset, column constraints ensure that there are no holes on the hull surface and the surface does not intersect itself. The feasibility rate is calculated as the proportion of samples in the generated table data $X'$ that meet the column constraints, compared to the total number of generated samples. The feasibility rate (ranging from 0 to 1) indicates the degree to which the generated table $X'$ adheres to the column constraints. The higher value signifies the greater adherence. Please refer to the appendix section for detailed metrics calculations and constraint rules.

Table 2 reveals that the TabDDPM strategy only achieved half of the generated data meeting the constraints. However, the DDPM with classifier resulted in all generated data satisfying the column constraints. In the diffusion model’s sample step, the inclusion of extra HI and AI led to a minor decrease in the feasibility rate by 0.02 and 0.08, respectively. The slight decline is acceptable, considering the model’s closer approximation to real-world data due to the inclusion of downstream task-oriented.

**Table 2:** The feasibility rate on the Ship-D dataset.

Strategy	Feasibility rate
RTVAE	0.179
CTGAN	0.018
TabDDPM	0.51
DDPM with classifier	1
ShipGen	0.98
TDGGD	0.92

Therefore, our model exhibited a high success rate in generating feasible design vectors $X'$ within complex tabular data spaces. The decline in feasibility satisfaction rate is attributable to the lack of feasibility consideration during sample generation and the added complexity from the HI and AI strategies. To enhance the success rate and performance, further work could focus on hyperparameter tuning, thereby achieving a higher success rate in generating feasible and high-performance data.

#### Coverage and realism

To quantify the statistical similarity, coverage and realism metrics are utilized between $(X, X')$, $(Y, Y')$, and (*Y*, $\tilde{Y'}$): An effective data generation model should aim to maintain a lower coverage rate for the generated data $X'$ while maximizing the realism of the predicted $Y'$. It requires a balanced optimization of these two metrics in the model training process. For a thorough breakdown of the coverage and realism calculations, please refer to the attachment.

The experimental results on are shown in Table 3, the our framework provides better data coverage and balanced realism and privacy protection.

**Table 3:** The coverage and realism on the Ship-D and California House dataset.

-	-	Ship-D				California House			
Methods	Data	Coverage	Realism	$\overline{\text {Converage}}$	$\overline{\text {Realism}}$	Coverage	Realism	$\overline{\text {Converage}}$	$\overline{\text {Realism}}$
RTVAE	$X,X'$	7.4574	4.9556	0.6547	0.5358	161.0204	3.0778	0.9947	0.8101
RTVAE	$Y,Y'$	2.0623	35.5565	0.9702	0.6172	80.6699	28.2033	0.9975	0.8454
RTVAE	$Y,\tilde{Y}$	2.0295	2.5623	0.6966	0.5606	171.4188	1.2100	0.9947	0.8389
CTGAN	$X,X'$	7.6455	5.2936	0.6296	0.6088	14.7484	4.4478	0.9993	0.9822
CTGAN	$Y,Y'$	1.2089	4.0176	0.9783	0.9380	23.0101	34.0267	0.9992	0.8942
CTGAN	$Y,\tilde{Y}$	1.7268	2.0848	0.8661	0.6171	10.5772	2.2568	0.9995	0.9911
TabDDPM	$X,X'$	10.6569	4.5891	0.6406	0.5566	59.2422	3.1655	0.998	0.8504
TabDDPM	$Y,Y'$	1.3926	0.2712	0.9804	0.7844	61.4435	12.5347	0.9981	0.8824
TabDDPM	$Y,\tilde{Y}$	4.3212	3.2444	0.6181	0.5971	57.2525	1.3514	0.9982	0.8773
DDPM with classifier	$X,X'$	12.1152	4.2084	**0.6675**	0.4714	234.6213	2.2474	*0.9930*	0.5340
DDPM with classifier	$Y,Y'$	2.5562	0.1843	***0.9659***	0.5186	250.0662	12.4111	***0.9931***	0.5400
DDPM with classifier	$Y,\tilde{Y}$	3.0122	2.0105	0.9212	0.6639	245.2265	1.0337	0.9932	0.6124
ShipGen	$X,X'$	11.4824	4.2776	**0.7687**	0.4843	165.6726	2.2640	*0.9950*	0.5359
ShipGen	$Y,Y'$	2.3382	0.2159	***0.9690***	0.7459	175.9302	11.9092	***0.9950***	0.5402
ShipGen	$Y,\tilde{Y}$	4.9948	3.6121	0.854	0.3846	172.0487	0.9720	0.9951	0.7116
TDGGD	$X,X'$	12.209	4.4451	**0.7005**	0.4700	1422.9737	38.0973	*0.9605*	0.2968
TDGGD	$Y,Y'$	2.4267	0.2834	***0.9699***	0.7157	6903.9999	5829.55	***0.9612***	0.2044
TDGGD	$Y,\tilde{Y}$	2.5232	2.1278	0.6886	0.5898	1366.1891	2.7569	0.9640	0.6216

The best results are marked as bold values in X data and bolditalics values in Y data.

For the Ship-D dataset, the TDGGD shows relatively high coverage and realism for the *X* data. It implies that the generated data effectively cover the distribution range of the original data while maintaining high realism. However, these results may not directly reflect the effectiveness of privacy protection. In terms of standardized metrics, the similarity in coverage for *X* ranges from 0.66 to 0.70, while the coverage for downstream task *Y* is around 96%, indicating a high coverage rate. Therefore, the coverage rate of *X* using the TDGGD (0.76) is lower than that of the ShipGen (0.70). It suggests that under the condition of predicting a similar *Y*, the TDGGD generates $X'$ with less similarity. The Realism metric also reflects a similar trend. Therefore, our framework achieve the better data coverage and balanced realism and privacy protection. The higher normalized coverage value indicates that fake data of TDGGD can effectively cover the distribution range of the original data, aiding in maintaining the utility of data for analysis and machine learning tasks. Although the normalized realism value is not the highest, it is actually an advantage for privacy protection. Excessively high realism could lead to generated data being too close to the original data, increasing the risk of leaking privacy information. Therefore, the TDGGD offers a balanced approach that maintains a certain level of realism while avoiding overexposure of original data characteristics.

For the California House dataset, Across all three data comparison(*X*, *Y*, $\tilde{Y}$), the normalized coverage values of the TDGGD are relatively low (especially extremely low in the *Y* ). It indicates that in terms of covering the distribution of the original data, TDGGD may not perform as well as others. For *X* and $\tilde{Y}$ data comparison, the normalized realism values of TDGGD are medium or slightly high relative to other methods. However, for the *Y* , the values are very low, indicating a significant difference between the generated $Y'$ data and the original *Y* data. Therefore, TDGGD achieve reduced over-similarity and balanced coverage and realism. The extremely low normalized realism values in the *Y* data suggest a substantial difference in characteristics between the generated and original data, which could aid in protecting privacy by reducing direct correlations between the generated and original data. Although TDGGD may not be the best in terms of coverage, its performance in realism (particularly in *X* and $\tilde{Y}$) indicates that it can preserve certain data characteristics while protecting privacy.

In summary, the framework provides a more balanced solution for privacy protection in Ship-D data compared to other strategies. It maintains data coverage and utility while appropriately controlling the realism of the data to reduce the risk of privacy breaches. In the California House dataset, the framework focuses more on reducing direct similarities between data, thus enhancing privacy protection. Although it might come at the cost of sacrificing data coverage, such an approach may be more suitable for applications with higher privacy protection requirements.

#### Scaled factor

The most crucial aspect of tabular data generation is measuring the impact of fake data using on downstream task predictions ($X'\xrightarrow {m}Y'$ or $X'\xrightarrow {f}\tilde{Y'}$ ). Please consult the attached document for a comprehensive explanation of the scaled factor metric calculations. The entire experiments’ results are also shown in appendix.

**Table 4:** Two dimensions results comparison $|1-scaledfactor|$ : the TDGGD between ($X'\xrightarrow {m}Y'$) and ($X'\xrightarrow {f}\tilde{Y'}$); the predictions between ($X \rightarrow Y$) with ($X'\xrightarrow {f}\tilde{Y'}$) on the TDGGD and ShipGen.

Target column	$Y'$	$\tilde{Y'}$	Difference	Ratio	EI+HI+AI	EI+HI	Difference	Ratio
Cw	0.3418	**0.0219**	– 0.3199	– 93.59%	***0.0219***	*0.3958*	0.3739	94.47%
SA1	**0.2430**	0.3621	0.1191	49.01%	***0.3621***	*0.5202*	0.1581	30.39%
SA2	**0.4589**	0.7742	0.3153	68.71%	***0.7742***	*1.2183*	0.4441	36.45%
Vol1	**0.2273**	**0.2239**	-0.0034	-1.50%	***0.2239***	*0.5620*	0.3381	60.16%
Vol2	**0.2031**	0.2499	0.0468	23.04%	***0.2499***	*0.3930*	0.1431	36.41%
MB	**0.0434**	0.2017	0.1583	364.75%	***0.2017***	*0.2262*	0.0245	10.83%
GC	**0.1397**	0.9516	0.8119	581.17%	***0.9516***	*1.9513*	0.9997	51.23%
Mean	**0.2367**	0.3979	0.1611	141.66%	0.3979	0.7524	0.3545	45.71%

The best results are marked as bold values in X data and bolditalics values in Y data.

The entire results on entire methods can be found as Supplementary Tables S7 and S8.

**Table 5:** Results comparison $|1-scaledfactor|$ between ($X'\xrightarrow {m}Y'$) and ($X'\xrightarrow {f}\tilde{Y'}$) on the MedHouseVal column of California House.

Strategy	$Y'$	$\tilde{Y'}$	Difference	Ratio (%)
RTVAE	0.1312	0.1182	– 0.0130	– 9.90
CTGAN	0.1941	0.0960	– 0.0981	– 50.54
TabDDPM	0.6701	0.4416	– 0.2285	– 34.10
DDPM with classifier	0.5031	0.7569	0.2538	50.45
ShipGen	0.4693	0.5847	0.1154	24.59
TDGGD	0.2660	0.9496	0.6836	256.99

The entire results can be found as Supplementary Tables S9 and S10.

From Table 4, only in the MB column does the TDGGD outperform all other strategies in terms of the realism of the predicted *Y*, indicating that for most target columns, the $|1-scaledfactor|$ values of the TDGGD are significantly higher than the other three strategies. From the data fidelity perspective, these higher values suggest a greater divergence between the generated data and the original data, which might be detrimental to the data’s practicality. More comprehensively, we constructed Table 4 to analyze the results of the TDGGD in both model prediction ($X'\xrightarrow {m}Y'$) and simulation calculation ($X'\xrightarrow {f}\tilde{Y'}$), leading to the following observations: For the Cw column, the $|1-scaled factor|$ value for $Y'$ (0.3418) is significantly higher than for $\tilde{Y'}$ (0.0219), indicating that the data generated using the simulation calculation is more similar to the original data. However, the $|1-scaled factor|$ for $Y'$ are smaller than $\tilde{Y}$ on the SA1, SA2, Vol2, MB, GC, Mean columns, meaning the data generated by the model prediction is closer to the original data in most cases. For Vol1, the $|1-scaled factor|$ values for both strategies are very close, suggesting that the similarity between the data generated by both downstream task ways and the original data is not significantly different in the Vol1 target column. Therefore, when applying the TDGGD, using the model prediction generally produces data more similar to the original *Y* data in Ship-D task. The simulation calculation strategy is more suitable in certain specific cases for generating results closer to the original data like Califronia House task.

From Table 4, in terms of the scaled factor between *Y* and $\tilde{Y'}$, the TDGGD shows a significant improvement over ShipGen. More comprehensively, on the Cw column, the $|1-scaledfactor|$ value of TDGGD (0.0219) is much lower than that of ShipGen (0.3958) with 0.3739 difference, indicating that the TDGGD is closer to the original data in the target column. On the SA1, SA2, Vol1, Vol2, MB, GC columns, the $|1-scaledfactor|$ values of the TDGGD method are generally lower than those of the ShipGen, indicating that TDGGD is closer to the original data in these target columns. On Mean columns, the TDGGD (average $|1-scaledfactor|$ value of 0.3979) is more similar to the original data than the ShipGen (average $|1-scaledfactor|$ value of 0.7524), with an average difference of 0.3545. Across all target columns, the average ratio of similarity to the original data for the TDGGD is 45.71%. It means that the $|1-scaledfactor|$ values of the TDGGD are approximately half of those of the ShipGen on Mean, indicating that its utility is closer to the original data.

Following the same strategy methods and comparisons on California House dataset, we lead to conclusions similar to above conclusion derived from the Ship-D dataset in Table 5. ($X'\xrightarrow {m}Y'$) are closer to ($X \rightarrow Y$), compared to ($X'\xrightarrow {f}\tilde{Y'}$). Our framework show a significant improvement in predicting target columns for downstream tasks.

#### Extra advantage: privacy protection

Similar with the privacy metrics in TabDDPM^3^, we adopt Distance to Closest Record (DCR) and Nearest Neighbour Distance Ratio (NNDR). Unlike de-identified data, which is susceptible to inference attacks, there is no straightforward one-to-one test between real data *X* and fake data $X'$. However, data science practitioners might employ intuitive and quantifiable metrics to measure the differences between them, such as Distance to Closest Record (DCR) and Nearest Neighbour Distance Ratio (NNDR). The detailed metrics calculations can be found at the appendix section.

The experimental results are shown in Tables 6 and 7. The TDGGD provides an excellent balance in terms of privacy protection, especially in the dispersion and diversity of generated data. The results demonstrate that our framework outperforms the other methods in safeguarding privacy.

For the Ship-D dataset, across the entire dataset ($X+X'$), the TDGGD yielded a DCR value of 5.3116, lower than the ShipGen but higher than both the TabDDPM and DDPM with classifier. It suggests that the TDGGD excels in maintaining data dispersion, which is beneficial for privacy protection. Specifically, for the fake data $X'$, the DCR value of TDGGD is the lowest (4.9025), indicating the best data dispersion, thus favoring privacy protection. Besides, the TDGGD has an NNDR value of 0.8326, slightly lower than the DDPM with classifier, but higher than both the TabDDPM and ShipGen. For the fake data $X'$, the NNDR value of TDGGD is 0.7959, which is lower than the TabDDPM and DDPM with classifier, but higher than the ShipGen. It indicates that the TDGGD, while maintaining data consistency, also reduces privacy risks. For the California House dataset, across the entire dataset ($X+X'$), the TDGGD exhibits the highest DCR value (1.3312) among all methods. Particularly for the fake data $X'$, its DCR value (0.0085) is significantly lower than other methods, suggesting that the distance between fake data $X'$ and the real data *X* is very small. It proximity reduces the likelihood of individual data points being identified, thereby enhancing privacy protection. Regarding the NNDR metric, the overall dataset ($X+X'$) performance of the TDGGD is 0.9406, substantially higher than other methods. It implies that in the TDGGD, the data points exhibit higher similarity, which could potentially increase privacy risks to some extent. However, for the fake data $X'$, the NNDR value of TDGGD (0.2777) is the lowest, indicating higher diversity in the generated data and thereby contributing to privacy protection.

**Table 6:** The DCR and NNDR on Ship-D and California House datasets.

Dataset	Strategy	DCR			NNDR		
		X+X’	X	X’	X+X’	X	X’
Ship-D	RTVAE	4.6912	3.8366	4.9217	0.8401	0.8589	0.8680
	CTGAN	4.7435	3.8366	4.7464	0.8434	0.8589	0.8049
	TabDDPM	5.2821	3.8366	6.1570	0.7905	0.8589	0.9127
	DDPM with classifier	5.3773	3.8366	6.0015	0.8359	0.8589	0.9066
	ShipGen	5.7998	3.8366	5.9438	0.7985	0.8589	0.7443
	TDGGD	5.3116	3.8366	4.9025	0.8326	0.8589	0.7959
California	RTVAE	0.5973	0.2549	0.2442	0.6304	0.6041	0.4051
House	CTGAN	0.5181	0.2549	0.4870	0.6013	0.6041	0.5715
	TabDDPM	0.8066	0.2549	0.8986	0.4961	0.6041	0.5116
	DDPM with classifier	0.8815	0.2549	1.6245	0.4924	0.6041	0.6026
	ShipGen	1.1297	0.2549	1.3020	0.6071	0.6041	0.6393
	TDGGD	1.3312	0.2549	0.0085	0.9406	0.6041	0.2777

**Table 7:** The five privacy metrics on Ship-D and California House datasets.

Datasets	Method	kAnonymization		lDiversityDistinct		kMap	DeltaPresence	IdentifiabilityScore	
		gt	syn	gt	syn	Score	Score	Score	socre_OC
Ship-D	RTVAE	999	43	999	43	31	42.52	0.0232	0.0298
	CTGAN	43	39	43	39	22	2.36	0.3710	0.3490
	TabDDPM	3	6	3	6	3	3.33	0.5400	0.0400
	DDPM with classifier	3	2	3	2	1	9.00	0.3200	0.0500
	ShipGen	3	4	3	4	2	5.00	0.4100	0.0300
	TDGGD	3	2	3	2	1	8.00	0.4200	0.0100
California House	RTVAE	1	3	1	3	5	3.20	0.0230	0.1180
	CTGAN	1	1	1	1	3	3.72	0.3190	0.1180
	TabDDPM	1	1	1	1	4	3.17	0.3200	0.0600
	DDPM with classifier	1	3	1	3	2	9.50	0.1900	0.0500
	ShipGen	1	1	1	1	1	2.60	0.1900	0.0300
	TDGGD	1	1	1	1	1	0.90	0.0100	0.0010

Thus, our framework demonstrates its advantages in privacy protection primarily. Compared to other methods, TDGGD shows superior privachy performance in the generated data X’. Our framework improved dispersion makes it more challenging to identify specific original data from the generated dataset. The low NNDR values indicate that the data X’ generated by the TDGGD possesses higher diversity. Our framework reduces the similarity between data points, thereby elevating the level of privacy protection. So TDGGD achieve better dispersion and high diversity.

### Summary

Our experimental results provide a clear and compelling analysis of how our novel diffusion model framework and constraint modeling techniques outperform the baseline models. By incorporating downstream task targets, the model is adept at producing data that is not only realistic but also aligned with specific application requirements. The approach ensures that the generated data is not only useful but also directly beneficial for downstream tasks, thereby increasing the practical value of our model. By leveraging the novel diffusion model framework and the efficient constraint modeling, we are able to augment datasets with high-quality, realistic fake data. This not only enriches the dataset but also enhances the robustness and performance of our model, especially in scenarios where tabular data scarcity is a significant concern.

## Discussion

### Convergence and early stopping

Our model’s convergence is determined by monitoring the gradient’s magnitude during training, which correlates with the loss function’s rate of descent. A very small gradient indicates that the model has likely reached a point of minimal loss, suggesting convergence. We use Early Stopping to prevent overfitting. This strategy is based on the model’s performance on a validation set, halting training if no significant improvement is observed after a set number of iterations. This approach not only prevents overfitting but also optimizes computational resources by avoiding unnecessary training beyond the point of diminishing returns.

### Diffusion timestep *T*

The diffusion timestep *T* is a critical hyperparameter in our model. It dictates the pace at which noise is incrementally introduced and then removed during the forward and reverse processes, respectively. The forward process turns tabular data into normal noise in the timestep*T* , and the reverse process reconstructs this process . The optimal value of *T* is influenced by various factors, including tabular data scales, computational constraints, and desired model performance. Our experiments utilized a timestep of $T=1000$, as referenced in previous work. We recognize that different settings for *T* might improve performance. We recommend more tests and adjustments to check this out. Additional details on hyperparameter settings are available in the Supplementary Material.

### Implications for future research

The findings from our experiments suggest that both convergence conditions and the choice of diffusion timestep T are pivotal in achieving optimal model performance. Future studies may gain from a closer look at how various data sets and computing conditions could impact the best choices for these parameters. Moreover, exploring adaptive methods for dynamically adjusting the timestep during training could provide further insights into enhancing model efficiency and accuracy.

## Conclusions

This study presents an innovative tabular data generation framework, termed TDGGD, integrating cutting-edge downstream task optimization techniques. Our framework emerges at the intersection of advanced machine learning models and large-scale data processing, reflecting the surge in demand for data-driven strategies and AI-readiness in numerous industries. The TDGGD approach showcases superior efficacy in generating tabular data with intricate inter-column dependencies, which is instrumental for simulating realistic databases. This study not only pushes the frontier in tabular data synthesis but also enriches the toolkit for overcoming complex data generation challenges, especially in AI-heavy sectors. Looking into the future, we aim to expand our investigation into diverse data modalities and a broader range of downstream applications, potentially including real-time decision-making scenarios and interactive AI systems.

## Supplementary Information

Supplementary Information.

## Data Availability

The datasets analyzed during the current study are available in the GitHub repository, including the California House dataset and the Ship Parameters dataset, which can be found at https://github.com/sonarsushant/California-House-Price-Prediction and https://github.com/noahbagz/ShipD, respectively.
