# Supplementary material for: A tabular data generation framework guided by downstream tasks optimization
Source: Sci Rep. 2024 Jul 3;14:15267. doi: 10.1038/s41598-024-65777-9 (PMC11757725; doi:10.1038/s41598-024-65777-9)
Supplement: Supplementary file 1 — Supplementary Information. [file 41598_2024_65777_MOESM1_ESM.pdf]

Supplementary material

Words Describe

As illustrated in Table 1, existing literature in this field has employed various terminologies to describe the process of generating table data. For the sake of consistency in expression throughout this research, this paper adopts the terminology from the first row of Table 1, which describes the process as "fake data using real data by generating tabular data".

Generate data	Real data	Fake data
Create data	Raw data	Synthetic data
Produce data	Source data	Simulated data
Generate information	Primary data	Artificial data
Fabricate data	Initial data	Fictitious data
Manufacture data	Base data	Fabricated data
Formulate data	Input data	Generated data
Compose data	Existing data	Dummy data
Derive data	Foundational data	Mock data
Construct data	Authentic data	Pseudo data

Table 1. Glossary of words with the same meaning in different situations.

DDPM Progress

DDPM (Denoising Diffusion Probabilistic Models) is a generative model based on diffusion processes, particularly suitable for image generation. The core idea of DDPM is to gradually introduce noise to transform data from a simple distribution, such as a Gaussian distribution, into a more complex data distribution, and then train a model to reverse this process, that is, to recover the original data from the noisy data. This process mainly consists of two steps: the forward diffusion process and the reverse denoising process.

1. Forward Diffusion Process: Starting from a data point, noise is gradually added, and after multiple time steps, the data ultimately becomes noise. This process is a deterministic Markov chain, and each step can be precisely described by mathematical formulas.

2. Reverse Denoising Process: This process is learnable, with the goal of reconstructing the original data from the noisy data. In practice, neural networks (such as UNet) are commonly used to predict the noise added at each step and subtract the predicted noise from the noisy data to gradually recover the original image.

The advantage of DDPM is its high-quality generated images and theoretically excellent properties. However, due to its Markov chain-based nature, the sampling process of DDPM can be relatively slow, which is one of the issues currently being addressed in research.

Since the proposal of DDPM, it has been applied to various image generation tasks and has achieved state-of-the-art results in some tasks. In addition, the principles of DDPM have also been used to develop more efficient image generation models, such as DALL-E and Stable Diffusion, etc.

The forward process $q(x_{1:T}|x_0) = \prod_{t=1}^{T} q(x_t|x_{t-1})$ gradually adds noise to an initial sample x_0 from the data distribution $q(x_0)$ sampling noise from the predefined distributions $q(x_t|x_{t-1})$ with variances $\{\beta_1, ..., \beta_T\}$.

The reverse diffusion process $p(x_{0:T}) = \prod_{t=1}^{T} p(x_{t-1}|x_t)$ gradually denoises a latent variable $x_T \sim q(x_T)$ and allows generating new data samples from $q(x_0)$. Distributions $p(x_{t-1}|x_t)$ are usually unknown and approximated by a neural network with parameters θ. These parameters are learned from the data by optimizing a variational lower bound:

$$\log q(x_0) \geq \mathbb{E}_{q(x_0)}\Big[\underbrace{\log p_\theta(x_0|x_1)}_{L_0} - \underbrace{KL(q(x_T|x_0)\,|q(x_T))}_{L_T} - \sum_{t=2}^{T}\underbrace{KL(q(x_{t-1}|x_t,x_0)\,|p_\theta(x_{t-1}|x_t))}_{L_t}\Big] \tag{1}$$

Gaussian DDPM operate in continuous spaces ($x_t \in \mathbb{R}^n$) where forward and reverse processes are characterized by Gaussian distributions:

$$q(x_t|x_{t-1}) := \mathcal{N}\left(x_t; \sqrt{1-\beta_t}x_{t-1}, \beta_t I\right)$$
$$q(x_T) := \mathcal{N}(x_T; 0, I)$$
$$p_\theta(x_{t-1}|x_t) := \mathcal{N}(x_{t-1}; \mu_\theta(x_t,t), \Sigma_\theta(x_t,t))$$

Using diagonal $\Sigma_\theta(x_t,t)$ with a constant σ_t and computing $\mu_\theta(x_t,t)$ as a function of x_t and $\varepsilon_\theta(x_t,t)$:

$$\mu_\theta(x_t,t) = \frac{1}{\sqrt{\alpha_t}}\left(x_t - \frac{\beta_t}{\sqrt{1-\bar{\alpha}_t}}\varepsilon_\theta(x_t,t)\right)$$

where $\alpha_t := 1-\beta_t,\ \bar{\alpha}_t := \prod_{i\leq t}\alpha_i$ and $\varepsilon_\theta(x_t,t)$ predicts a "groundtruth" noise component ε for the noisy data sample x_t. In practice, the objective (1) can be simplified to the sum of mean-squared errors between $\varepsilon_\theta(x_t,t)$ and ε over all timesteps t:

$$L_t^{simple} = \mathbb{E}_{x_0,\varepsilon,t}\|\varepsilon-\varepsilon_\theta(x_t,t)\|_2^2 \tag{2}$$

The training and sampling phase can be shown as follow:

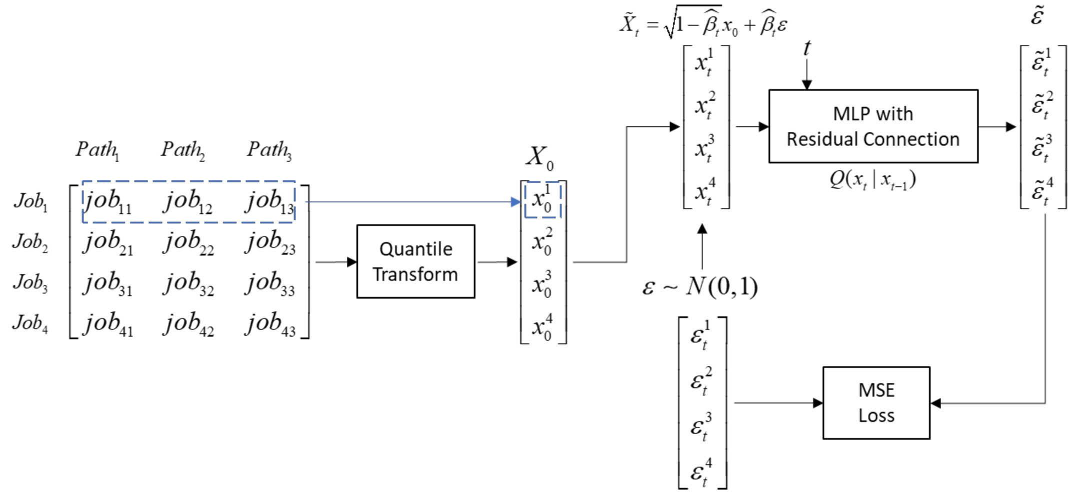

Figure 1. In the training phase, the DDPM model was utilized to predict a denoising step, which was conditioned on a given timestep and noise level. The model was fed with an input consisting of three independent and identically distributed (i.i.d.) paths, each simulating a distinct job, totaling four jobs in the simulation.

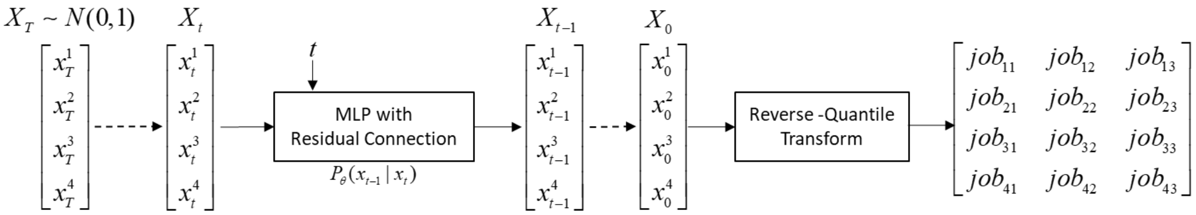

Figure 2. During the sampling phase, the DDPM model denoise a sequence of vectors across multiple timesteps, effectively reconstructing the underlying signal and generating new, noise-free data points."

The TDGGD method is based on DDPM, which add EI, AI and HI in training and sampling phase for improving downstream task. The extra modules are shown in Figure 1 of origin manuscript.

Process of data flow with notation

When confronted with large-scale tabular data consisting of real numbers, researchers often encounter a significant challenge: how to effectively utilize the data while ensuring its privacy. This necessitates the introduction and implementation of privacy protection strategies. In the absence of such strategies, those who hold or collect the data (referred to as upstream data holders or collectors) would directly share the data X with downstream tasks. This data, denoted as $m \times n$ real tabular data $X = [c_i] = [r_j]$ (where $i \in [1,n]$ and $j \in [1,m]$), comprises m-dimensional vectors for each column c_i and n-dimensional vectors for each row r_j. Consequently, this data remains in its raw and unprocessed state for downstream tasks.

However, the growing emphasis on privacy protection necessitates more than mere data sharing without any protective measures. As a result, privacy-preserving strategies for generating tables have been introduced. Within this approach, upstream

data holders or collectors retain the real data X while generating fake data X' through the use of table generation models. Although these fabricated data exhibit structural and feature-wise similarities to the original data, they do not contain any information that can directly identify or infer individual privacy.

Consequently, downstream tasks are now provided with the generated fake data X' instead of the original real data X. They can employ the fabricated data to predict the target variable Y' through machine learning model or calculating the target variable $\tilde{Y}$ using mathematical formulas. As fake data Y' has undergone privacy protection, the downstream tasks are unable to extract any information about individual privacy.

Data Prepare for EI, HI, AI

Figure 3 in manuscript illustrates the process of constructing training data for the supervised models used in our approach. For the EI module, which employs a supervised binary classification model, the correct class input X is assigned a predicted label of 1, while the incorrect class input X' is assigned a predicted label of 0. In contrast, the HI and AI modules utilize supervised regression models, where the regression input X yields output labels for ci and five target labels, respectively.

In machine learning, supervised binary classification and regression are two common task types. A binary classification model aims to predict a binary outcome, typically 0 or 1, based on input features, indicating whether a sample belongs to a certain class. A regression model, on the other hand, predicts a continuous outcome, usually a real number. Training these models requires labeled data, where each input sample corresponds to a known output label, enabling the model to learn the mapping from inputs to outputs.

The EI module adopts a supervised binary classification model, where each input sample X is associated with a label indicating its class. A correct prediction is labeled as 1, and an incorrect one as 0, allowing the model to learn to differentiate between correct and incorrect input samples.

In comparison, the HI and AI modules employ supervised regression models, where each input sample X is associated with one or more continuous values as output labels. For the HI module, the output label is ci, and for the AI module, there are five target labels. This enables the model to learn how to predict these continuous output values based on input features.

To train these models, a corresponding training dataset is necessary, where each sample includes input features and the corresponding output labels. These training data are typically obtained through manual or automatic annotation. During training, the model attempts to minimize the discrepancy between predicted outputs and actual labels, thus learning the mapping from inputs to outputs.

Therefore, Figure 3 is a crucial step in preparing the training data for the construction of the index. It visualizes how the tabular data X is structured to create labels for the HI, EI, and AI modules. Consequently, there are no specific requirements for downstream tasks regarding the model's architecture. The model incorporates multiple modules—HI, EI, and AI—for instance, in Experiment 1, we trained a total of 1 HI, 12 EI, and 3 AI neural network modules, each corresponding to the supervised inputs (data and label) as detailed in Table 3 of manuscript.

Compared Methods

1. RTVAE: the state-of-the-art variational auto-encoder for tabular data generation[1].

2. CTGAN: the conditional generative adversarial network which can handle tabular data[2].

3. Base strategy: the standard TabDDPM[3].

4. EI strategy: DDPM with classifier[4], that is, the main pipeline use DDPM and introduce the easy indicator as guidance.

5. EI+HI strategy: similar with ShipGen[5], that is, the DDPM with easy indicator and hard indicator.

6. EI+HI+AI strategy: The implementation of the framework proposed in this article, that is, the DDPM with easy indicator, hard indicator and ambiguous indicator.

Hyper-Parameters

Table 2. The main pipline of DDPM's parameters

Parameter Variable	Variable Value	Meaning
xdim	X.shape[1]	Dimension of parametric design vector
datalength	X.shape[0]	Number of samples
X_LL	limits['Lower Limits']	Lower limits of parametric design vector variables
X_UL	limits['Upper Limits']	Upper limits of parametric design vector variables
ydim	Y.shape[1]	Number of objectives
cdim	Cons.shape[1]	Number of classes for classifier
gamma	0.5	Weight of feasibility guidance for guided sampling
lambdas	[1, 1, 1, 1, 1]	Dummy variables for performance guided sampling
tdim	128	Dimension of latent variable
net	[1024, 1024, 1024, 1024]	Network architecture
batch size	1024	Batch size
Training Epochs	100000	Number of training epochs
Diffusion Timesteps	1000	Number of diffusion timesteps
lr	0.00025	Learning rate
weight decay	0.01	Weight decay
device name	'cuda:0'	GPU device name

Table 3. The easy indicator's parameters

Parameter Variable	Variable Value	Meaning
xdim	X.shape[1]	Dimension of parametric design vector
cdim	Cons.shape[1]	Number of classes in the classifier
tdim	128	Dimension of latent variable
net	[64, 64, 64]	Network architecture
Training Epochs	150000	Number of training epochs
device name	'cuda:0'	GPU device name

Table 4. The hard indicator's parameters

Parameter Variable	Variable Value	Meaning
xdim	X.shape[1]	Dimension of parametric design vector
ydim	1	Trains regression model for each objective
tdim	256	Dimension of latent variable
net	[1024, 1024, 1024]	Network architecture
Training Epochs	10000	Number of training epochs
num regressors	Y.shape[1]	Number of regressors to train
Model Labels	Y.columns.values.tolist()	Labels for regressors
lr	0.001	Learning rate
weight decay	0.01	Weight decay
device name	'cuda:0'	GPU device name

Table 5. The ambiguous indicator's parameters

Parameter Variable	Variable Value	Meaning
xdim	X.shape[1]	Dimension of parametric design vector
ydim	1	Number of objectives per regression model
tdim	256	Dimension of latent variable
net	[1024, 1024, 1024]	Network architecture
Training Epochs	10000	Number of training epochs
num ambiguous	X.shape[1]	Number of regressors to train
Model Labels	[List specific labels]	Labels for regressors
lr	0.001	Learning rate
weight decay	0.01	Weight decay
lambdas	[List specific values]	Dummy variables for performance guided sampling

Results from all experiments.

Training Results

Table 6. The HI performance had a high goodness-of-fit with training dataset on Ship-D and California House

Hard on California House		Hard on Ship-D	
Target Column	R2 score	Target Column	R2 score
Target mean	0.9893	Cw	0.9878
Target std	0.9803	SA1	0.9921
Target max	0.9678	SA2	0.9914
Target min	0.9957	Vol1	0.9903
MedHouseVal	0.9879	Vol2	0.9898
-	-	MB	0.8308
-	-	GC	0.9320

Table 7. The AI performance had a high goodness-of-fit with training dataset on Ship-D and California House.

Ambiguous on California House			
Target Column	R2 score	Target Column	R2 score
MedInc	0.9751	Population	0.9594
HouseAge	0.9512	AveOccup	0.9553
AveRooms	0.9822	Latitude	0.9895
AveBedrms	0.9547	Longitude	0.9856
Ambiguous on Ship-D			
Target Column	R2 score	Target Column	R2 score
Lb	0.9888	SK_z	0.9925
Ls	0.9907	Kappa_stern	0.9887
Bd	0.9897	Adel stern	0.9991
Dd	0.9923	Bdel stern	0.9991
Bs	0.9870	Beta trans	0.9915
WL	0.9907	Bc trans	0.9892
Bc	0.9912	Rc Trans	0.9890
Beta	0.9949	Rk trans	0.9879
Rc	0.9897	bit_BB	1.0000
Rk	0.9888	bit_SB	1.0000
Abow	0.9986	Lbb	0.9999
Bbow	0.9988	Hbb	1.0000
BK_z	0.9870	Bbb	1.0000
Kappa_bow	0.9899	Lbbm	0.9942
Adel bow	0.9990	Rbb	0.9998
Bdel bow	0.9989	Kappa_SB	0.9999
Adrft	0.9988	Lsb	0.9994
Bdrft	0.9990	HSBOA	0.9999
Cdrft	0.9909	Hsb	0.9999
bit_EP_S	0.9994	Bsb	0.9998
bit_EP_T	0.9993	Lsbm	0.9939
Atrans	0.9854	Rsb	0.9999

Sampling Results

Feasibility

All strategies are trained on the 30,000 feasible samples from the Ship-D dataset to implicitly learn the combinations of parameter values that define "feasibility." Since the California Housing dataset lacks explicit column constraints, the section focuses solely on the Ship-D dataset with clear constraints. For example, the values of the Lb and Ls columns in the Ship-D dataset are clearly constrained: $0.05 < Lb < 0.9$, $0.0 < Ls < 0.9$, and $Lb + Ls < 1$. The complete column constraints can be found in the Ship-D appendix[6]. The section generated 1,000 samples and evaluated the dataset coverage and feasibility of these samples.

DCR and NNDR

Distance to Closest Record (DCR) is a metric that measures the average distance between all samples and its nearest neighbor, whether from the real data X or the fake data X'. It helps us understand the spatial distribution and density of data points. When DCR is low, it indicates that data points are closer to each other, suggesting a dataset with strong spatial correlations. Conversely, a high DCR suggests that data points are more dispersed, indicating a dataset with weaker spatial correlations. The process involves calculating the distance between a specific data point and every other point. Then the smallest value among these distances is identified. The value signifies the distance from the data point to its closest neighboring point.

The calculation formula is $\text{DCR} = \min(distance_i)$, where $i = 1, 2, \ldots, n$, and $distance_i$ is the distance between the data point and other data points i. Using the Euclidean distance, the distance between data points i and j can be expressed as:

$$distance_{ij} = \sqrt{(x_i - x_j)^2 + (y_i - y_j)^2} \tag{3}$$

where (x_i, y_i) and (x_j, y_j) represent the spatial coordinates of data points i and j, respectively.

Nearest Neighbour Distance Ratio (NNDR) is a metric that measures the ratio of a data point's distance with its nearest neighbor to the distance with its second-nearest neighbor. It is used to determine whether a data point is located in a clustered area within the dataset. Specifically, if a data point is in a uniformly distributed dataset, the distance between its nearest and second-nearest neighbors should be relatively large. Conversely, if a data point is in a clustered area of the dataset, the distance

between its nearest and second-nearest neighbors should be relatively small. Therefore, by calculating the ratio of the distances to the nearest and second-nearest neighbors, one can infer the density of the area where the data point is located.

The formula for calculating is NNDR $= \frac{distance_1}{distance_2}$, where $distance_1$ is the distance to the nearest neighbor, and $distance_2$ is the distance to the second-nearest neighbor. A smaller NNDR indicates that the data point is in a clustered area, while a larger NNDR suggests that the data point is in a relatively dispersed area.

Coverage and Realism

Coverage is an measurement of the extent to which a set of generated samples X', represents the numerical distribution of the original training data X. For the real tabular data X, a lower coverage value is preferable as it indicates a more focused range of generated data, concentrated in effective latent space regions, thereby reducing susceptibility to privacy attacks. For the downstream prediction target Y, a higher coverage value is advantageous as it suggests that the generated data do not interfere with downstream task predictions.

Realism assesses the extent to which the generated samples X', replicate the distribution of the real data X. For generated fake data X', a lower realism value is preferable, implying a significant divergence between the generated and real data distributions, thus enhancing data security. Conversely, for the downstream prediction target Y, a higher realism value is beneficial as it indicates minimal interference of the generated data with downstream task predictions.

For fairness comparing on different range, we normalized coverage and realism values ($\overline{\text{Converage}}$ and $\overline{\text{Realism}}$). The standardized metrics offer more consistent and comparable measurements.

Scaled Factor

The scaled factor refers to the difference between the prediction targets Y on the training dataset X and the prediction targets Y' on the synthetic dataset X'. The metric clearly illustrates the extent to which generated data affects downstream tasks. When the scaled factor equals 1, Y equals Y', indicating that the generated data has no interference. Therefore, $|1 - scaledfactor|$ column is added for the distance from 1. The larger its value, the greater the difference between Y and Y'. The larger signifies a more significant impact of the generated fake data on the prediction targets. The formula for scaled factor is given by $\frac{1}{n}\sum_{i=1}^{n}\frac{\text{real Y}}{\text{fake Y'}}$.

Table 8. The sacled factor between $X \rightarrow Y$ and $X' \xrightarrow{m} Y'$ on Ship-D target columns

Strategy	Target Column	μ_Y	σ_Y	$\mu_{Y'}$	$\sigma_{Y'}$	scaledfactor	$\lvert 1 - scaledfactor \rvert$
RTVAE	Cw	-73.4045	17.3776	40.5999	45.2801	-0.5858	1.5858
RTVAE	SA1	-1.7150	0.5348	-0.0249	0.8666	0.0147	0.9853
RTVAE	SA2	-1.0860	0.4451	0.2662	0.7349	-0.2416	1.2416
RTVAE	Vol1	4.7820	0.8084	-2.3730	3.1526	-0.5105	1.5105
RTVAE	Vol2	3.8043	0.6154	-1.2207	2.3319	-0.3322	1.3322
RTVAE	MB	-0.4072	0.0994	0.0131	0.2104	-0.0365	1.0365
RTVAE	GC	2.4361	0.5295	1.6623	1.0587	0.6725	0.3275
CTGAN	Cw	-73.4045	17.3776	-15.3715	32.0597	0.2166	0.7834
CTGAN	SA1	-1.7150	0.5348	-0.7619	0.9167	0.4178	0.5822
CTGAN	SA2	-1.0860	0.4451	-0.4185	0.6487	0.3890	0.6110
CTGAN	Vol1	4.7820	0.8084	1.2700	2.2683	0.2725	0.7275
CTGAN	Vol2	3.8043	0.6154	1.4040	1.5312	0.3794	0.6206
CTGAN	MB	-0.4072	0.0994	-0.0756	0.2093	0.2075	0.7925
CTGAN	GC	2.4361	0.5295	1.7353	0.8562	0.6977	0.3023
Base	Cw	-73.4045	17.3776	-73.8871	15.7326	1.0687	0.0687
Base	SA1	-1.7150	0.5348	-1.6807	0.5483	0.9477	0.0523
Base	SA2	-1.0860	0.4451	-1.0635	0.4463	1.0458	0.0458
Base	Vol1	4.7820	0.8084	4.7979	0.7492	1.0456	0.0456
Base	Vol2	3.8043	0.6154	3.8051	0.5832	1.0438	0.0438
Base	MB	-0.4072	0.0994	-0.4195	0.0681	1.0450	**0.0450**
Base	GC	2.4361	0.5295	2.5362	0.5413	1.0786	0.0786
EI	Cw	-73.4045	17.3776	-73.8431	8.3935	1.0715	0.0715
EI	SA1	-1.7150	0.5348	-1.7271	0.3947	0.9649	0.0351
EI	SA2	-1.0860	0.4451	-1.0640	0.2830	1.0270	0.0270
EI	Vol1	4.7820	0.8084	4.5438	0.3894	0.9880	0.0120
EI	Vol2	3.8043	0.6154	3.6253	0.2837	0.9893	0.0107
EI	MB	-0.4072	0.0994	-0.4549	0.0397	1.1384	**0.1384**
EI	GC	2.4361	0.5295	2.4854	0.1717	1.0562	0.0562
EI+HI	Cw	-73.4045	17.3776	-71.5708	8.4342	1.0334	0.0334
EI+HI	SA1	-1.7150	0.5348	-1.5506	0.3709	0.8608	0.1392
EI+HI	SA2	-1.0860	0.4451	-0.9498	0.2633	0.9023	0.0977
EI+HI	Vol1	4.7820	0.8084	4.4225	0.3984	0.9578	0.0422
EI+HI	Vol2	3.8043	0.6154	3.5280	0.2889	0.9601	0.0399
EI+HI	MB	-0.4072	0.0994	-0.4502	0.0483	1.1202	**0.1202**
EI+HI	GC	2.4361	0.5295	2.5762	0.4036	1.1004	0.1004
EI+HI+AI	Cw	-73.4045	17.3776	-92.7744	17.9744	1.3418	0.3418
EI+HI+AI	SA1	-1.7150	0.5348	-2.2131	0.3943	1.2430	0.2430
EI+HI+AI	SA2	-1.0860	0.4451	-1.5045	0.3577	1.4589	0.4589
EI+HI+AI	Vol1	4.7820	0.8084	5.6398	0.9767	1.2273	0.2273
EI+HI+AI	Vol2	3.8043	0.6154	4.4045	0.6724	1.2031	0.2031
EI+HI+AI	MB	-0.4072	0.0994	-0.4154	0.0609	1.0434	**0.0434**
EI+HI+AI	GC	2.4361	0.5295	2.6820	0.2354	1.1397	0.1397

Table 9. The sacled factor between $X \rightarrow Y$ and $(X' \xrightarrow{f} \tilde{Y}')$ on Ship-D target columns

Strategy	Target Column	μ_Y	σ_Y	$\mu_{\tilde{Y}'}$	$\sigma_{\tilde{Y}'}$	scaledfactor	$\|1 - scaled factor\|$
RTVAE	Cw	-73.4045	17.3776	-42.9994	39.5392	0.6159	0.3841
RTVAE	SA1	-1.7150	0.5348	-0.1044	0.9444	0.0588	0.9412
RTVAE	SA2	-1.0860	0.4451	0.4474	0.8454	-0.4135	1.4135
RTVAE	Vol1	4.7820	0.8084	2.3708	2.3684	0.5059	0.4941
RTVAE	Vol2	3.8043	0.6154	1.6339	1.8416	0.4400	0.5600
RTVAE	MB	-0.4072	0.0994	-0.3335	0.1483	0.9028	0.0972
RTVAE	GC	2.4361	0.5295	0.0320	2.0582	0.0023	0.9977
CTGAN	Cw	-73.4045	17.3776	-72.6662	30.1342	1.0403	0.0403
CTGAN	SA1	-1.7150	0.5348	-0.5613	1.0186	0.3065	0.6935
CTGAN	SA2	-1.0860	0.4451	-0.3130	0.7299	0.2860	0.7140
CTGAN	Vol1	4.7820	0.8084	4.4476	1.8323	0.9528	0.0472
CTGAN	Vol2	3.8043	0.6154	3.1434	1.2505	0.8497	0.1503
CTGAN	MB	-0.4072	0.0994	-0.3898	0.1488	1.0578	0.0578
CTGAN	GC	2.4361	0.5295	0.0727	1.6755	0.0098	0.9902
Base	Cw	-73.4045	17.3776	-49.5252	40.2674	0.7155	0.2845
Base	SA1	-1.7150	0.5348	-0.3752	1.0011	0.2225	0.7775
Base	SA2	-1.0860	0.4451	0.1972	0.9377	-0.1515	1.1515
Base	Vol1	4.7820	0.8084	3.2038	2.3383	0.7072	0.2928
Base	Vol2	3.8043	0.6154	2.1506	1.6878	0.6021	0.3979
Base	MB	-0.4072	0.0994	-0.3756	0.1458	0.9306	0.0694
Base	GC	2.4361	0.5295	-1.8673	1.5114	-0.7919	1.7919
EI	Cw	-73.4045	17.3776	-45.1235	28.2738	0.6631	0.3369
EI	SA1	-1.7150	0.5348	-1.3000	1.0896	0.7222	0.2778
EI	SA2	-1.0860	0.4451	-0.3586	0.8536	0.3442	0.6558
EI	Vol1	4.7820	0.8084	2.6136	1.5203	0.5722	0.4278
EI	Vol2	3.8043	0.6154	2.7707	1.4864	0.7556	0.2444
EI	MB	-0.4072	0.0994	-0.3705	0.1653	0.9300	0.0700
EI	GC	2.4361	0.5295	2.5374	2.3416	1.0755	0.0755
EI+HI	Cw	-73.4045	17.3776	-42.2757	29.2377	0.6042	0.3958
EI+HI	SA1	-1.7150	0.5348	-0.8977	1.1652	0.4798	0.5202
EI+HI	SA2	-1.0860	0.4451	0.1741	0.8724	-0.2183	1.2183
EI+HI	Vol1	4.7820	0.8084	2.0609	1.4394	0.4380	0.5620
EI+HI	Vol2	3.8043	0.6154	2.2730	1.4066	0.6070	0.3930
EI+HI	MB	-0.4072	0.0994	-0.4972	0.1493	1.2262	0.2262
EI+HI	GC	2.4361	0.5295	-2.2838	1.4452	-0.9513	1.9513
EI+HI+AI	Cw	-73.4045	17.3776	-67.7135	46.0376	0.9781	0.0219
EI+HI+AI	SA1	-1.7150	0.5348	-1.1182	1.1431	0.6379	0.3621
EI+HI+AI	SA2	-1.0860	0.4451	-0.2159	1.1018	0.2258	0.7742
EI+HI+AI	Vol1	4.7820	0.8084	3.5454	2.3842	0.7761	0.2239
EI+HI+AI	Vol2	3.8043	0.6154	2.7390	1.7258	0.7501	0.2499
EI+HI+AI	MB	-0.4072	0.0994	-0.4759	0.1576	1.2017	0.2017
EI+HI+AI	GC	2.4361	0.5295	0.1183	1.6842	0.0484	0.9516

Table 10. The sacled factor between $X \rightarrow Y$ and $(X' \xrightarrow{f} Y')$ on California House target columns. The original target is MedHouseVal column

Strategy	μ_Y	σ_Y	$\mu_{Y'}$	$\sigma_{Y'}$	scaledfactor	$\|1 - scaled factor\|$
RTVAE	2.0686	1.1540	1.9772	0.8843	1.1312	0.1312
CTGAN	2.0686	1.1540	2.0895	0.9865	1.1941	0.1941
Base	2.0686	1.1540	2.0599	1.0466	1.6701	0.6701
EI	2.0686	1.1540	1.8660	0.6202	1.5031	0.5031
EI+HI	2.0686	1.1540	1.8333	0.6705	1.4693	0.4693
EI+HI+AI	2.0686	1.1540	0.9197	0.1300	0.7340	0.2660

Table 11. The sacled factor between $X \rightarrow Y$ and $(X' \xrightarrow{f} \tilde{Y}')$ on California House target columns. The original target is MedHouseVal column

Strategy	μ_Y	σ_Y	$\mu_{\tilde{Y}'}$	$\sigma_{\tilde{Y}'}$	scaledfactor	$\lvert 1 - scaledfactor \rvert$
RTVAE	2.0686	1.1540	1.9543	0.8721	1.1182	0.1182
CTGAN	2.0686	1.1540	1.9184	0.9901	1.0960	0.0960
Base	2.0686	1.1540	1.7697	1.1220	1.4416	0.4416
EI	2.0686	1.1540	2.1671	1.0866	1.7569	0.7569
EI+HI	2.0686	1.1540	1.9710	1.0190	1.5847	0.5847
EI+HI+AI	2.0686	1.1540	2.4272	0.9901	1.9496	0.9496

DataSet

Table 12. The Ship-D dataset's target columns or prediction label.

Symbol	Meaning	Calculation Formula
C_w	Aggregated sum of wave drag coefficients	$\frac{R_w}{\frac{1}{2}\rho U^2 \cdot \mathrm{LOA}^2}$
$\mathrm{SA}_{50\%}$	Surface area of the hull up to 50% of its total depth	$\log_{10}\left(\frac{\int_0^{T/D_d=0.5} \delta SA(z)\,\delta z}{LOA^2}\right)$
$\mathrm{SA}_{100\%}$	Total surface area of the hull	$\log_{10}\left(\frac{\int_0^{T/D_d=1.0} \delta SA(z)\,\delta z}{LOA^2}\right)$
$\mathrm{Vol}_{50\%}$	Displaced volume of the hull up to 50% of its total depth	$-\log_{10}\left(\frac{\int_0^{T/D_d=0.5} \delta V(z)\,\delta z}{\mathrm{LOA}^3}\right)$
$\mathrm{Vol}_{100\%}$	Total displaced volume of the hull	$-\log_{10}\left(\frac{\int_0^{T/D_d=1.0} \delta V(z)\,\delta z}{\mathrm{LOA}^3}\right)$
$\mathrm{Vol}_{\mathrm{MaxBox}}$	Volume of the MaxBox	$-\frac{\mathrm{Volume}_{\mathrm{MaxBox}}}{\mathrm{Volume}_{T/D_d=1.0}}$
GC	Gaussian curvature	$\log_{10}(GC \cdot LOA^2)$

Privacy Metrics

In privacy section, we adopts four popular metrics.

1. kAnonymization: the k-anon ratio between the real data and the synthetic data.

For each dataset, it is computed the value k which satisfies the k-anonymity rule: each record is similar to at least another k-1 other records on the potentially identifying variables. lDiversityDistinct the distinct l-diversity ratio between the real data and the synthetic data. For each dataset, it computes the minimum value l which satisfies the distinct l-diversity rule: every generalized block has to contain at least l different sensitive values. We simulate a set of the cluster over the dataset, and we return the minimum length of unique sensitive values for any cluster.

2.kMap: the minimum value k that satisfies the k-map rule.

The data satisfies k-map if every combination of values for the quasi-identifiers appears at least k times in the reidentification(synthetic) dataset.

3.DeltaPresence: the maximum re-identification probability on the real dataset from the synthetic dataset.

For each dataset partition, we report the maximum ratio of unique sensitive information between the real dataset and in the synthetic dataset.

4.IdentifiabilityScore: the re-identification score on the real dataset from the synthetic dataset.

We estimate the risk of re-identifying any real data point using synthetic data. Intuitively, if the synthetic data are very close to the real data, the re-identification risk would be high. The precise formulation of the re-identification score is given in the reference below. Reference: Jinsung Yoon, Lydia N. Drumright, Mihaela van der Schaar, "Anonymization through Data Synthesis using Generative Adversarial Networks (ADS-GAN): A harmonizing advancement for AI in medicine," IEEE Journal of Biomedical and Health Informatics (JBHI), 2019. Paper link: https://ieeexplore.ieee.org/document/9034117

References

1. Liu, T., Qian, Z., Berrevoets, J. & van der Schaar, M. GOGGLE: Generative modelling for tabular data by learning relational structure. In The Eleventh International Conference on Learning Representations (2023).
2. Zhao, Z., Kunar, A., Birke, R. & et al. Ctab-gan: Effective table data synthesizing. In Asian Conference on Machine Learning, 97–112 (PMLR, 2021).
3. Kotelnikov, A., Baranchuk, D., Rubachev, I. & Babenko, A. Tabddpm: Modelling tabular data with diffusion models. In International Conference on Machine Learning, 17564–17579 (PMLR, 2023).

4. Dhariwal, P. & Nichol, A. Diffusion models beat gans on image synthesis. In Advances in Neural Information Processing Systems, vol. 34, 8780–8794 (2021).

5. Bagazinski, N. J. & Ahmed, F. Shipgen: A diffusion model for parametric ship hull generation with multiple objectives and constraints. J. Mar. Sci. Eng. **11**, 2215 (2023).

6. Bagazinski, N. J. & Ahmed, F. Ship-d: Ship hull dataset for design optimization using machine learning. arXiv preprint arXiv:2305.08279 (2023).
